# Annotations

**Published:** 1887-08-27

**Authors:** 


### Annotations.

**Registered Plumbers.** The worst duly qualified medical practitioner is infinitely better for all-round practice than the cleverest "quack." If the registration of adequately examined and licensed men does nothing else, it secures for the public a certain minimum amount of knowledge and trained capacity. There is a level below which the registered practitioner cannot fall. In certain branches of service for the public it has been found necessary in the public interest to frame and enforce regulations which are a decided infringement of individual liberty. The legal, medical, and to some extent the clerical professions are hedged round with restrictions which act as barriers to outsiders. All this is done purely in the interests of the body of the people, and can only be justified on the ground of high public expediency. It is proposed to extend similar regulations to plumbers, and for precisely similar reasons. In large towns plumbers have functions of the very first order of importance. The whole system of public and private drainage, water-supply, gas-service, and other various work is entirely in their hands. A moment's reflection will show the vast influence on the general well-being which those persons have who make and mend our drains, our water-pipes, and our gas-fittings. The adulteration of food and drink is hardly a question of greater importance than those of drainage and water and gas-supply. Good drainage is one of the indispensable conditions of health in large cities. Without it a moderate standard of physical well-being is impossible; whilst with bad drainage a whole city may be given over to all kinds of infectious diseases, and to a death-rate three or four times greater than it would otherwise be. At the present time many plumbers seem to have neither brains nor consciences. If registration be made compulsory we shall at least secure a certain amount of more or less educated brains, and it may reasonably be hoped that with these there will be a corresponding development of conscience.

**Sea Water for Inland Towns.** Mr. Ellis Lever, a well-known resident in Manchester, writes to the *Manchester Examiner*, suggesting the many advantages which would result to that city from a constant and plentiful supply of sea water. The city, which has the reputation of being the rainiest in England, has lately suffered exceedingly from the prolonged drought. Mr. Ellis Lever is of opinion that sea water might be advantageously employed for baths, closets, drains, and all kinds of domestic purposes other than drinking, as well as for motor power. It is probable that the use of sea water for baths, closets, and drains would be highly serviceable in London and other large cities. The abundance of sodium chloride and other antiseptic salts in solution in it, would not only destroy the offensive effluvia given off by drainage, but, if used in sufficient quantity, would render sewage to a large extent innocuous. It would be very easy to bring to London any quantity of sea water every day. If that were done, vast swimming-baths might also be constructed for public use at no very great cost, and with the most beneficial results. The larger London becomes, the more urgent is it that the question of the health of the people should be considered in a broad, liberal, and scientific spirit. A little rock will split a very large ship, and such an uninteresting and, to some people, unimportant a subject as drainage, may be the means of destroying whole cities of industrious and energetic people. Intelligence and foresight are as necessary as diligence and honesty.

**Swimming Baths.** It is a pity that mayors and town councillors are so often chosen from among the uninstructed classes. Many of our towns and cities have populations and institutions as large and important as those of the smaller European States. How seldom do we find among their citizens men of public spirit and of a high order of intelligence! No one will dispute the proposition that it is important to preserve among all classes of our people a good physique; and it is equally beyond question that our town-bred populations are small, feeble, and insignificant creatures compared with the corresponding classes in the country. Whilst squires and rich merchants will take no end of trouble in the improvement of their cattle, their plants, and their flowers, who among them has the sense or the patriotism to spend a thought or a sovereign on the development of their fellow human beings? Fighting men may be wanted some day, and what kind of fighters would the men of our narrow streets and crowded houses make? Narrow streets and crowded houses seem to be unavoidable in large cities; but at least such measures as are easily within reach should be employed to counteract their injurious influence. Every large town should have gymnasia in every quarter of it, and large swimming-baths erected at the public expense, and placed under public control. The citizens of all ages and ranks should be encouraged by all possible means to avail themselves of these means of recreation and physical development. We are aware that such preaching is often addressed to deaf ears; but so important is the subject, that those who see it in its true light should take every opportunity of proclaiming their convictions upon the house-tops, in order that sooner or later a public opinion may be created which shall be strong and intelligent enough to deal effectually with all these questions. What is everybody's business is nobody's business; but if some more than usually intelligent mayor desires to signalise his term of office by useful and enduring public services, he cannot do better than devote his leisure to the study of the best methods of physical development. Other mayors will soon follow his example.

